# Building genomically‐informed demographic models to guide management of invasive hybrids

**DOI:** 10.1002/eap.70116

**Published:** 2025-10-23

**Authors:** Robert D. Cooper, Arianne F. Messerman, Christopher A. Searcy, Erin Toffelmier, Gregory F. Grether, H. Bradley Shaffer

**Affiliations:** ^1^ Department of Ecology and Evolutionary Biology University of California Los Angeles California USA; ^2^ La Kretz Center for California Conservation Science, Institute of the Environment and Sustainability, University of California Los Angeles California USA; ^3^ Missouri Department of Conservation, Central Regional Office Columbia Missouri USA; ^4^ Department of Biology University of Miami Coral Gables Florida USA; ^5^ Present address: Department of Fish and Wildlife Conservation, Virginia Polytechnic Institute and State University Blacksburg Virginia USA

**Keywords:** *Ambystoma californiense*, amphibian, California tiger salamander, conservation, demography, hybridization, integral projection model, invasive species

## Abstract

Invasive species present one of the most challenging threats to native biodiversity, particularly when they hybridize with imperiled native taxa. In California, hybridization between the endangered California tiger salamander (“CTS,” *Ambystoma californiense*) and the invasive barred tiger salamander (“BTS,” *Ambystoma mavortium*) is one of the best understood examples of this management challenge. Reclusive life history and cryptic hybridization, often on private land, render eradication programs difficult or impossible. This study evaluates hydroperiod management as a tool to conserve and maintain native CTS populations threatened by hybridization. We adapt a recent, empirically informed Bayesian integral projection model (IPM) for CTS to incorporate new results that link genotype and ecology to fitness, and use this individual‐based model to evaluate alternative management scenarios. We found overwhelming support for the importance of hydrology in both native and hybrid populations, where a 10‐day increase in hydroperiod can increase population growth rate (λ) 17% and triple the carrying‐capacity (*K*). We assess hydroperiod management as a strategy to control and contain hybrid introgression, and suggest a three‐pronged strategy. First, for native populations not at risk of hybridization, hydroperiod should be increased to >120 days to support robust populations. Second, within the geographic hybrid zone, hydroperiod should be reduced to limit hybrid populations, maintain vernal pool function, and improve the efficiency of adult hybrid removal. Finally, our models indicate that managers should combine hydroperiod management with rapid field‐based genotyping and hybrid removal, focusing on ponds where hybrids are rare, typically at the leading edge of the hybrid swarm. Efforts should also prioritize high‐intensity surveys and early removal as opposed to long‐duration (10+ years), lower effort surveys. This study demonstrates the value of integrating demographic, genetic, and ecological information to evaluate strategies for endangered species management, and may serve as modeling framework for a wide variety of imperiled species.

## INTRODUCTION

Effective wildlife conservation requires an understanding of the demographic processes that govern threatened populations. Adaptation of quantitative models, especially demographic models, can significantly improve conservation actions (García‐Díaz et al., 2019) because such models can be used to evaluate alternative management strategies for taxa that would otherwise be difficult to compare (Brooks, 2020; Chapron et al., 2003; Wiens et al., 2017). Though the accuracy of these models has sometimes been questioned (Beissinger & Westphal, 1998; Coulson et al., 2001; Ellner et al., 2002), other studies have demonstrated their value for comparing population responses to different ecological and management scenarios (Brook et al., 2000). When properly parameterized and applied, population models can be vital tools for making informed management decisions (Chaudhary & Oli, 2020), allowing researchers to evaluate inherent trade‐offs between strategies and identify optimal conservation outcomes (García‐Díaz et al., 2019).

In this study, we employ population models to evaluate specific recovery efforts of the endangered California tiger salamander (*Ambystoma californiense*; hereafter “CTS”). CTS are endemic to California, where they inhabit low‐ to medium‐elevation grasslands that characterized inland central California before western settlement. Individuals spend the majority of their lives in underground rodent burrows, from which they emerge during winter rain events to breed in temporary rain‐filled ponds (Trenham et al., 2000). Adults lay eggs that hatch into aquatic predatory larvae which typically require a minimum of 90 days to grow and complete metamorphosis (Johnson et al., 2013; Petranka, 1998); hydroperiods shorter than 90 days often result in mass larval mortality.

A number of threats contribute to the endangerment of this salamander, including habitat loss (particularly agricultural conversion of grasslands), invasive species (Fisher & Shaffer, 1996), and climate change (Searcy & Shaffer, 2016). One of the most complex issues impeding CTS recovery is hybridization with an introduced congener, the barred tiger salamander (*Ambystoma mavortium*; hereafter “BTS”; Fitzpatrick et al., 2010; Riley et al., 2003). After its intentional introduction in the 1950s into the Salinas Valley (Monterey County, CA), non‐native BTS have become established, and their range has significantly expanded from the initial introduction sites through migration and colonization (Fitzpatrick et al., 2010), although not as much as previously believed (Fitzpatrick et al., 2024). Non‐native BTS produce fertile hybrids with CTS, and their offspring often exhibit greater fitness than either parental genotype (Burger et al., 2024; Cooper & Shaffer, 2021, 2024; Fitzpatrick & Shaffer, 2007b; Johnson & Johnson, 2010), though recent data suggest that there may be metabolic costs to this apparent hybrid vigor in terrestrial life stages (Fitzpatrick et al., in review). Regardless, this larger and more fit hybrid larval phenotype threatens to replace the unique genetic diversity found in native CTS and degrade the endangered vernal pool community in which they develop (Ryan et al., 2009; Searcy et al., 2016). For these reasons, the USFWS identifies hybrid management as a top priority for the recovery of this species (U.S. Fish and Wildlife Service, 2017). It is therefore vital to develop and evaluate management solutions that reduce the success of non‐native hybrids in the field, while minimizing the impact on other native, often endangered, community members.

Hybrid success appears to be related to pond hydrology. Most agricultural/livestock ponds have been modified to extend their hydroperiod well beyond that of most natural vernal pools. Previous field surveys have found that non‐native allele frequencies are higher in these ponds with artificially extended hydroperiods (Fitzpatrick & Shaffer, 2007a). Subsequently, an experimental mesocosm study investigated the effect of hydroperiod on hybrid and native larval survival and mass at metamorphosis (Johnson et al., 2013), two demographic parameters essential to CTS ecology (Searcy et al., 2014, 2015). Johnson et al. (2013) found that longer hydroperiods strongly favor non‐native genotypes, while shortened hydroperiods favor native genotypes to a limited degree. Recent work employed 14 large (10‐meter diameter) constructed ponds situated at the edge of the CTS/BTS hybrid zone to evaluate this pattern in the most natural setting possible (Cooper & Shaffer, 2024). This multiyear experiment yielded mixed results: the short hydroperiod treatments reduced but did not eliminate the hybrid advantage and resulted in lower survival and mass at metamorphosis for all genotypes. While hydroperiod manipulation alone will not remove hybrids from the landscape, it may still be the most effective management tool available, particularly when combined with other approaches.

The present study builds on this experimental work to definitively evaluate hydroperiod management as a conservation tool in the hybrid CTS/BTS system. Here, we seek to answer three questions: (1) Can short hydroperiods at the lower limit of larval developmental time (80–90 days) support stable CTS populations? (2) Do short hydroperiods reduce hybrid success enough to slow or reverse increases in non‐native alleles? (3) Would targeted hybrid removal effectively reduce non‐native allele frequencies? To evaluate these critical conservation questions, we adapted an existing Bayesian integral projection model (IPM) for native CTS (Messerman et al., 2023). We expand on Messerman et al.'s (2023) model, modifying their IPM to incorporate estimates of larval survival and mass at metamorphosis conditional on individual genotype and breeding pond characteristics derived from our recent hydroperiod experiment (Cooper & Shaffer, 2024). The Bayesian framework of the original and our modified model enables us to integrate and interpret uncertainty from the numerous sources of empirical data, allowing us to evaluate our level of confidence at each stage in the model. From this modified IPM, we estimate the intrinsic population growth rate (λ), density‐dependent carrying capacity (*K*), and 100‐year population viability for 25 demographic scenarios spanning a range of hydroperiods and starting proportion of hybrids. We then model different hybrid removal strategies to help guide the development and implementation of this important management strategy. We believe this integration of genomic and ecological data in demographic simulations can serve as a framework for evaluating the management of many imperiled species.

## METHODS

### Individual‐based IPM

We adapted the CTS demographic model constructed by Messerman et al. (2022, 2023) to include traits critical to hybrid management at both individual (mass and genotype) and ecological (hydroperiod) scales. The original IPM leveraged multiple long‐term ecological studies (Searcy et al., 2014; Trenham et al., 2000) to predict CTS population dynamics given established relationships between demographic rates and body size. We expanded on this by constructing an individual‐based model that tracks an individual's phenotype (mass and survival) and projects shifts in these traits over time. Our individual‐based implementation of the IPM includes all demographic functions (listed in Table 1) from Messerman et al. (2023), unless otherwise stated. To examine the effects of hydroperiod and non‐native ancestry proportion (Hybrid Index Score; hereafter “HIS”; Johnson et al., 2010) on CTS demography, we augment the functions that predict mass at metamorphosis and larval survival using modified functions we derived from the constructed pond hydroperiod experiment (Cooper & Shaffer, 2024). These functions fit Bayesian regression models using custom scripts in the R package jagsUI (Kellner, 2015; R Core Team, 2021) to predict larval survival to metamorphosis and metamorph mass based on pond hydroperiod and individual HIS (see Appendix S1). These functions were applied to both density‐independent and density‐dependent versions of the IPM. We quantify uncertainty in the model by randomly drawing 500 values from the posterior distribution of each model parameter, then performing a simulation with each parameter set, resulting in 500 unique simulations (“model iterations”). This approach propagates the uncertainty from each Bayesian function through the entire simulation, such that variation in the resulting population statistics represents the uncertainty associated with each component function.

**TABLE 1:** The major steps in the density‐independent model.

Step	Model function	Description
1	Survival	Survival probability is estimated, and a binary state (alive or dead) is randomly drawn from the binomial distribution centered on this probability. Individuals that are greater than 15 years old are automatically coded as dead.
2	Growth	Growth is randomly selected from the predicted probability density distribution and used as the new mass.
3	Maturity	Maturity probability is estimated, and a binary state (mature or immature) is randomly drawn from the binomial distribution centered on this probability. Individuals that have reached maturity remain mature in all subsequent years (until death).
4	Fertility	Fertility is estimated from the new adult mass. Fertility that is less than zero is truncated to zero.
5	Breeding	Breeding probability is estimated, and a binary state (breeding or not breeding) is randomly drawn from the binomial distribution centered on this probability.
6	Death	All individuals that did not survive are removed from the data frame.
7	Select breeders	A separate data frame is created for males and females that are mature and breeding that year. In the model that includes environmental stochasticity, the number of females that breed is dependent on rainfall. If there are zero male or zero female breeders, then no offspring are created.
8	Pair breeders	Each female is randomly assigned one male as a mate. Each male may mate multiple times. All females breed, but not all males necessarily breed.
9	Larval survival	The offspring HIS and pond hydroperiod are used as parameters in the larval survival function determined from the hydroperiod experiment (Cooper & Shaffer, 2024).
10	Fecundity	Female fecundity, estimated as the product of fertility and larval survival.
11	Metamorphs	A new data frame is created with each row representing a new metamorph derived from female fecundity.
12	Metamorph HIS	The mid‐parent value (mean) of HIS is assigned to each offspring.
13	Metamorph mass	The mass at metamorphosis for each offspring is predicted using the HIS and hydroperiod as inputs into the metamorph mass model derived from the recent hydroperiod study (Cooper & Shaffer, 2024).

*Note*: All steps are repeated for each year the model is run. This is used as the basic model framework that is further augmented for other model implementations (i.e., density‐dependent and population viability analysis).

Abbreviation: HIS, Hybrid Index Score.

### Demographic scenarios

We included two treatments to construct several demographic scenarios: hydroperiod and proportion of hybrids. We evaluated five levels of the “hydroperiod” treatment, from 80 to 120 days in 10‐day intervals (80‐, 90‐, 100‐, 110‐, and 120‐day). These levels were chosen to replicate the previous hydroperiod study, which was used to parameterize key model functions (Cooper & Shaffer, 2024). The “proportion of hybrids” treatment consisted of populations with a specific starting proportion of hybrid individuals. We included five levels of hybrid proportions: 0, 0.25, 0.5, 0.75, and 1.0. For example, a population of 100 individuals with a 0.25 proportion of hybrids initially contains 25 hybrids and 75 natives that mate at random. A hybrid proportion of 0 or 1 indicates all‐native or all‐hybrid populations, respectively. The HIS of each hybrid was assigned using a beta‐binomial distribution with alpha = 10 and mu = 0.75. This distribution was chosen to simulate the skewed distribution of HIS observed in non‐native ponds, which have a right‐skewed central tendency with strong left‐hand tails. Native CTS were assigned an HIS of 0.05. This non‐zero value for native HIS reflects the average native HIS recorded in the previous hydroperiod study due to uncertainty in the HIS estimation (Cooper & Shaffer, 2024). This resulted in a total of 25 simulations per demographic model (5 hydroperiod levels × 5 hybrid proportions). All simulations focused on an exemplar pond with a volume of 1000 m^3^, which represents an average sized pond with a surface area between 1000 and 1500 m^2^, equivalent to a circular pond with a 36–44 m diameter.

### Density‐independent model and population growth rate (λ)

Density‐independent models are useful for comparing intrinsic growth rates across demographic scenarios. The intrinsic growth rate, or lambda (λ), describes population growth under “ideal” or low‐density scenarios, when density‐dependent factors are not operating. If λ is less than 1, the population decreases, if λ is greater than 1, the population increases; populations with greater λ grow more rapidly and are thus able to recover more rapidly from events that reduce population size.

The general model framework is outlined in Table 1. This model was run for 12 simulated years, representing roughly 4 generations given the 3.16‐year generation time in CTS (Messerman et al., 2023). This allowed sufficient time for the population to experience exponential growth, without requiring excessive computational time, which likewise grows exponentially. Each year, the combined number of juveniles and adults (*N*_*t*_) was recorded. Lambda was estimated as the slope of the log‐transformed *N*_*t*_ with respect to time from years 3 to 12 using the linear regression function “lm” in the R statistical language (R Core Team, 2021). Years 1–2 include transient dynamics that reflect the starting conditions and not true population growth and are therefore not used to calculate λ. This model was iterated 500 times to sample variation in the Bayesian posterior distributions of each model parameter.

### Density‐dependent model and carrying capacity

The density‐dependent model is identical to the density‐independent model except that it includes several population‐limiting modifications. The original CTS IPM by Messerman et al. (2023) incorporates the effect of egg density on two vital rates: larval survival and metamorph mass, both of which were estimated from field and mesocosm studies. Egg density was inferred from field data (Trenham et al., 2000) to serve as a proxy for larval density. The original IPM included log‐transformed egg density and log‐transformed larval survival, which exhibited a negative linear relationship: as egg density increases, larval survival decreases. Here, we instead center the function on the larval survival probability that is predicted using our hydroperiod and HIS‐based model. Therefore, larval survival predicted from the hydroperiod and HIS functions (see Appendix S1) represents survival at average egg density. We then use the slope determined from Messerman et al. (2023) to account for the increase in larval survival at low densities and decrease in survival at high densities.

The second density‐dependent function from Messerman et al. (2023) defined a negative linear relationship between log‐transformed egg density and log‐transformed mass at metamorphosis. We similarly adapted this relationship to our present study by re‐centering the predicted mass of a given metamorph on the value estimated from our HIS and hydroperiod functions (see Appendix S1). Thus, average egg density observed in the field would produce metamorph mass equal to our model predictions, and increases or decreases in egg density would result in smaller or larger individuals, respectively.

These density‐dependent simulations yield the estimated carrying capacity on which the population converges. The carrying capacity (*K*) can be estimated as the average population size (*N*_*t*_) once births and deaths reach an equilibrium, which occurred within 20 years in all pilot simulations. We imposed a maximum adult population size of 100,000 individuals to improve computational efficiency in all simulations except density independence. This threshold represents a sufficiently large upper limit for population size (i.e., 3‐fold larger than the population size predicted for Olcott Lake (Messerman et al., 2023), which is 100‐fold larger than the 1000 m^3^ pond simulated in this study). Simulations were run for 100 years, and we report *K* as the median adult population size from years 75 to 100 across the 500 model iterations.

### Population viability analysis

Environmental stochasticity was incorporated into the model to assess the long‐term viability of each demographic scenario. The amount of rainfall in a given year drastically affects the magnitude of CTS breeding and recruitment. Low‐rainfall years result in fewer adults emerging from upland refugia to breed (Trenham et al., 2000) and reduce offspring survival through metamorphosis (Searcy & Shaffer, 2016). Therefore, extended droughts may lead to significant population reduction and possibly extinction.

We adapted two functions from Messerman et al. (2023) to account for this environmental effect on population vital rates. First, we used the cumulative precipitation from December through January, the period when most females emerge to breed (Searcy & Shaffer, 2011), to determine the proportion of breeding females. We used the same climate data from the Vacaville and Nut Tree Airport Weather Stations that were used in Messerman et al. (2023) to maintain the potentially site‐specific empirical link between rainfall and recruitment and allow direct comparisons between models. Second, we used the cumulative precipitation from October through June to determine larval survival probability. Messerman et al. (2023) used empirical data to fit a three‐component, piecewise linear model that predicts the proportion of successful metamorphs given the October–June precipitation. This model includes two inflection points; the lower defines a level of rainfall (404.5 mm) below which there is complete reproductive failure, while the upper corresponds to the amount of rainfall (674.5 mm) above which all late‐stage larvae are predicted to metamorphose. Between these two points, the model predicts a linear increase in larval survival. It is important to note that these values were calculated for native CTS populations, and may differ slightly for hybrids (see *Discussion*).

Historical climate data were incorporated into the model to evaluate relative population viability given environmental stochasticity. We randomly sampled rainfall data, with replacement, for each year of the simulation. It is important to note that future climate change may impact population persistence (Messerman et al., 2023) and should be considered when evaluating absolute persistence probability. However, our goal was to compare the relative effects of different demographic scenarios on population persistence. Given this, we used historical data since it has a finer resolution and encompasses true annual variability. Each model iteration ran for 100 years, at which time the population size (*N*_100_) was recorded. Populations that dropped below the 3‐individual quasi‐extinction threshold used in a PVA based on the original CTS IPM (Messerman et al., 2023) were considered extinct. The population HIS was also recorded to track changes in the frequency of non‐native alleles. We estimated the stable population size at the end of the PVA as the median population size from years 75 to 100, well after birth and death rates reach equilibrium. The ratio between the stable population size and the carrying capacity, or “percent of *K*,” was estimated as $\left({\bar{N}}_{75-100}/K\right)\times 100\%$ for each demographic scenario.

### Hybrid removal simulation

Hybrid salamanders exhibit greater survival and fitness than all‐native CTS, even under short hydroperiods (Cooper & Shaffer, 2024). Therefore, containment and control of hybrid introgression will likely require strategies to identify and remove hybrid salamanders. Here, we modeled the demographic consequences of hybrid removal across a range of parameters that describe how the removal could be implemented. We define the following parameters: (1) BTS detection threshold (values simulated: 0.05–0.55 in increments of 0.05)—the average proportion of the genome that must contain non‐native alleles for the detection assay to correctly identify an individual as hybrid. In practice, this value is determined by the number of single nucleotide polymorphisms (SNPs) that are screened by the assay. (2) Probability of adult capture (0.45–1.0 in increments of 0.05)—the probability of capturing any adult that is present in a pond. This is a proxy for sampling effort: greater field effort results in a greater probability of detecting a hybrid individual. (3) Maximum number of salamanders screened (0–225 in increments of 25)—the maximum number of breeding adult salamanders that can be screened from a given pond. This may be limited based on the cost of genotyping assays or permitting restrictions. (4) Number of consecutive years hybrids are removed (5–60 in increments of 5 years)—the number of years at the beginning of the 100‐year simulation during which hybrids are screened and removed, after which no removal takes place. (5) Individual HIS threshold for removal (0.1–0.9 in increments of 0.1)—the threshold above which hybrids are removed. Higher threshold values relax the criteria for removal, resulting in a less stringent strategy with fewer hybrids removed. Note, this is the actual measured value of HIS, given the number of markers screened.

We simulated these removal parameters over a combination of hydroperiod (80, 90, 100, 110, and 120 days) and proportion hybrid (0.25, 0.75, and 1.00) scenarios using the mean value across 500 draws from the Bayesian model parameters' posterior distributions. Simulations were performed using the PVA model that includes environmental stochasticity. In total, we performed 124,200 hybrid removal simulations. We also performed 145,800 simulations without hybrid removal to establish a baseline population‐level HIS after 100 years. We then calculated the percent change in population HIS (∆HIS) for each scenario as:

$$\begin{array}{l} ∆\mathrm{HIS}=\frac{100\%\times \left({\mathrm{HIS}}_{\text{yesRemoval}}-{\mathrm{HIS}}_{\text{noRemoval}}\right)}{{\text{HIS}}_{\text{noRemoval}}} \end{array}$$

Negative values for ∆HIS indicate a beneficial decrease in non‐native alleles, whereas positive ∆HIS indicates a detrimental increase in non‐native alleles despite hybrid removal activities.

### Statistical methods

We explored the effects of hydroperiod and initial hybrid proportion on demographic parameters using models in the R statistical language (R Core Team, 2021). We modeled λ and *K* using linear mixed‐effect models and PVA “percent of *K*” using a generalized linear mixed‐effect model with binomial error distribution. We used the “model iteration” (1–500) as the random effect in each model, which represents the specific set of parameter values drawn from the Bayesian posterior of each underlying demographic function in the IPM such that all simulations with the same model iteration used the same set of values. For all models, we report the slope (β), 95% confidence interval, and *p* value for each predictor variable. All log‐linear dependent variables (*y*) were transformed by adding the smallest non‐zero value observed for *y* to each value of *y* before taking the natural log, to avoid undefined values produced by zeros. Model slopes for log‐linear models are reported without exponentiating. We report the percent increase in *y* with each change (*c*) in the independent variable, using the equation: $∆y=\left(e^{c\beta }-1\right)\times 100\%$. We then scale dependent variables using the scale function in R to compare their relative effect on *y* using the equation: $\frac{\beta 1}{\beta 2}$. Note that the scaling is based on the values selected for the simulations rather than observed SDs from natural populations.

## RESULTS

All results and model statistics are summarized in Table 2.

**TABLE 2:** Statistical model outputs for each test performed.

Parameter	Raw β	Scaled β	Upper CI	Lower CI	*p* value	Model type
Population growth rate (λ)						
Hydroperiod	1.66 × 10^−2^	0.235	1.65 × 10^−2^	1.67 × 10^−2^	<2 × 10^−16^	Log‐lmer
Proportion hybrid	0.260	0.092	0.255	0.264	<2 × 10^−16^	Log‐lmer
Carrying capacity (*K*)						
Hydroperiod	0.132	6.44	0.130	0.134	<2 × 10^−16^	Log‐lmer
Proportion hybrid	1.411	1.65	1.340	1.482	<2 × 10^−16^	Log‐lmer
PVA—Population size						
Hydroperiod	0.133	1.88	0.132	0.135	<2 × 10^−16^	Log‐lmer
Proportion hybrid	1.36	0.48	1.29	1.43	<2 × 10^−16^	Log‐lmer
PVA—Percent of *K*						
Hydroperiod	0.086	1.22	0.083	0.090	<2 × 10^−16^	Log‐lmer
Proportion hybrid	0.823	0.291	0.699	0.948	<2 × 10^−16^	Log‐lmer
PVA—Prob. of persistence						
Hydroperiod	0.406	5.75	0.384	0.430	<2 × 10^−16^	Logit‐glmer
Proportion hybrid	4.31	1.52	3.98	4.66	<2 × 10^−16^	Logit‐glmer

*Note*: Raw is the unscaled model estimate for each parameter. Scaled is the model estimate for parameters after standardization (mean = 0 and SD = 1).

### Population growth rate (λ)

**FIGURE 1:**
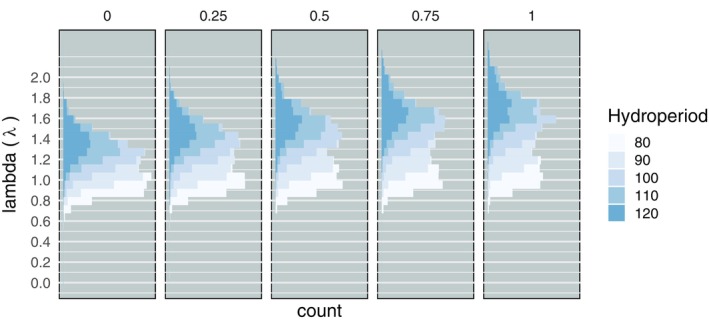
Density‐independent model estimates of population growth rate (Lambda or λ) across demographic scenarios. λ is estimated as the slope of the log‐normalized adult population size and time (in years), after an initial burn‐in of 2 years. The simulation was initiated with different combinations of pond hydroperiod (colors) and the starting proportion of hybrid individuals in the population (panels). Longer hydroperiods and higher hybrid proportions yield greater λ estimates. Short hydroperiods and more native populations result in lower values for λ, some of which are less than 1, indicating a declining population.

The simple density‐independent model yielded estimates of the intrinsic, per capita population growth rate (λ). Across 500 model iterations each of 25 different demographic scenarios, λ ranged widely from 0.004 to 2.308. Across all simulations, median λ was 1.31 (95% credible interval = 1.09, 1.53). In the all‐native simulations with the longest hydroperiod (120 days), conditions that best represent Olcott Lake where the original IPM was primarily developed, the median λ was 1.49 (95% CI = 1.28, 1.72). Lambda was significantly correlated with both hydroperiod (lmer: β = 1.66 × 10^−2^, confidence interval = [1.65 × 10^−2^, 1.67 × 10^−2^], *p* < 2 × 10^−16^; Figure 1 and Appendix S1: Figure S1) and the proportion of hybrids (lmer: β = 0.260, CI = [0.255, 0.264], *p* < 2 × 10^−16^; Figure 1). A 10‐day increase in pond hydroperiod increases λ by 17%, while a population with 25% more hybrids experiences a 6.5% increase. This pattern is more evident when the input variables are scaled and centered, where hydroperiod (β = 0.235) has a 2.6× greater effect on λ than the proportion of hybrids (β = 0.092). The scenarios that yielded a λ < 1 were mostly distributed in the short hydroperiods, with *n* = 1944 (77.8%), *n* = 453 (18.1%), *n* = 71 (2.8%), *n* = 16 (0.6%), and *n* = 11 (0.4%) populations having λ<1 in the 80‐, 90‐, 100‐, 110‐, and 120‐day hydroperiods, respectively (see Appendix S1). These declining populations most frequently had a lower proportion of hybrids, such that there were 763/2500 (30.1%) in the all‐native populations and only 309/2500 (12.4%) in the all‐hybrid populations with λ<1.

### Carrying capacity (*K*)

**FIGURE 2:**
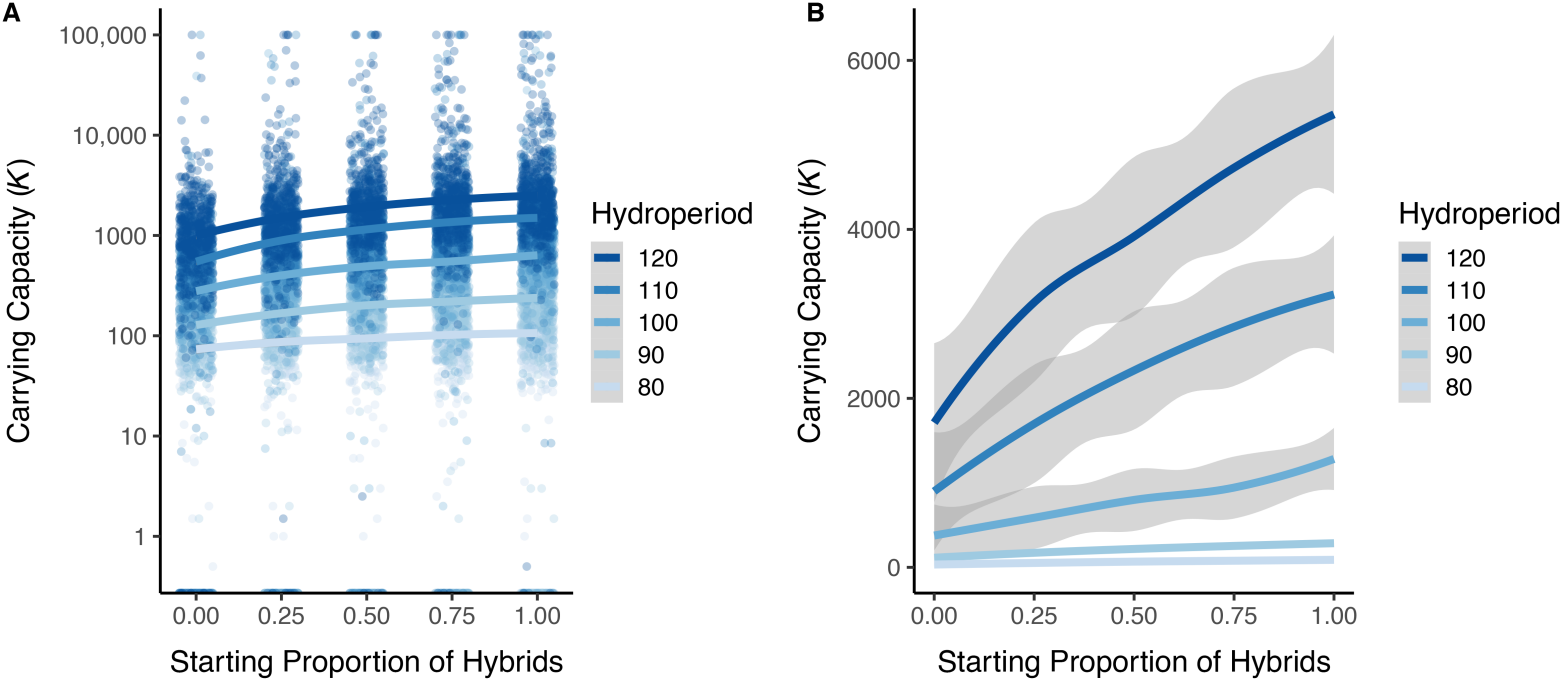
Density‐dependent model estimates for carrying capacity (*K*) across a range of pond hydroperiods and starting proportion of hybrids. Panels show the median population size from years 75 to 100 of the 100‐year simulations. The first 75 years are removed to allow the populations sufficient time to reach their stable equilibrium. Colored dots indicate values for each simulation, colored lines represent locally estimated scatterplot smoothing (loess) model predictions with SE depicted as gray ribbons. Panel (A) is plotted using a base 10 logarithmic scale to improve resolution, while panel (B) is plotted on a linear scale.

The density‐dependent model yielded different adult carrying capacity (*K*) estimates for each combination of hydroperiod and proportion hybrid initial conditions. Estimates of *K* ranged from 0 to 100,000 adults, which was the maximum population size allowed in the simulation (Figure 2 and Appendix S1: Figure S2). Both hydroperiod (log‐lmer: β = 0.132, confidence interval = [0.130, 0.134], *p* < 2 × 10^−16^; Figure 2) and the proportion of hybrids (log‐lmer: β = 1.411, CI = [1.340, 1.482], *p* < 2 × 10^−16^; Figure 2) significantly affected the estimate of *K*. For example, a 10‐day increase in hydroperiod resulted in a 273% increase in *K*, while a 25% increase in the initial hybrid proportion resulted in a 42% increase in *K*. When the predictors are scaled, hydroperiod (β = 6.44) had a 3.9× greater effect on *K* than the proportion of hybrids (β = 1.65). There were 1841 simulations that resulted in population collapse (i.e., *K* = 0), predominantly in the shorter hydroperiod range (*n* = 1181 [47.2%], *n* = 435 [17.4%], *n* = 149 [6.0%], *n* = 45 [1.8%], *n* = 31 [1.2%]), in the 80‐, 90‐, 100‐, 110‐, and 120‐day hydroperiods, respectively (Appendix S1: Figure S3). The number of failed populations exhibited a 2.4‐fold difference between all‐native 573/2500 (22.9%) and all‐hybrid 235/2500 (9.4%) populations (Figure 2 and Appendix S1: Figure S4).

### Population viability analysis

**FIGURE 3:**
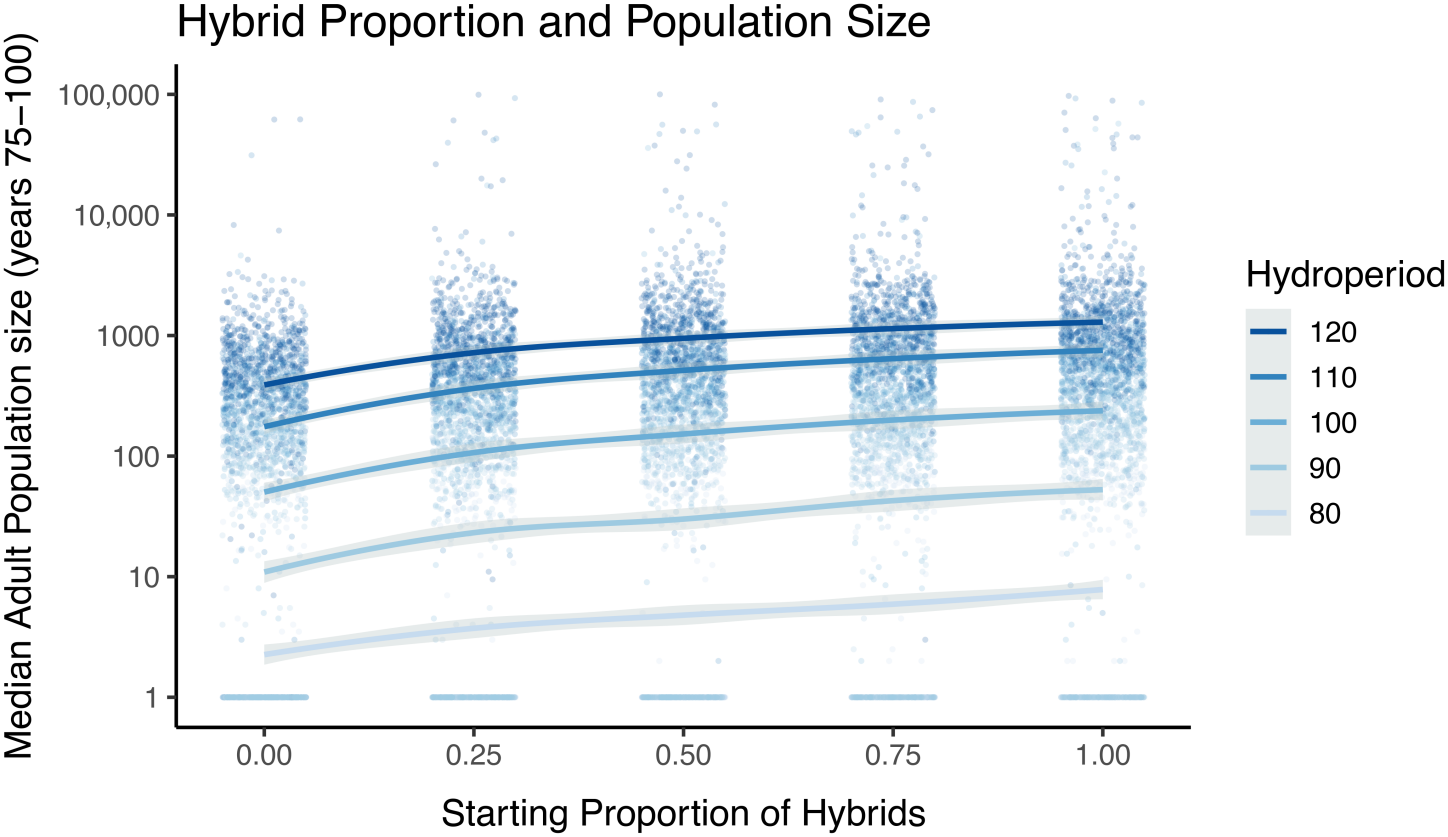
Population viability analysis (PVA) model estimates of stable population size. Population size is calculated as the median number of adults in the population in years 75–100. The PVA model includes environmental stochasticity, which yields new estimates for equilibrium population sizes. We show the estimated median population size with respect to the starting proportion of hybrids and hydroperiod (colors) simulated across 500 draws from the posterior distributions of all demographic functions (dots). Colored lines represent locally estimated scatterplot smoothing (loess) model predictions with SE depicted as gray ribbons. Population size is shown on a logarithmic scale to increase the resolution of low population‐size scenarios. Horizontal lines of points at the bottom of each panel represent populations that have gone extinct.

Population size at the end of the 100‐year simulation was positively correlated with hydroperiod (log‐lmer: β = 0.133, CI = [0.132, 0.135], *p* < 2 × 10^−16^; Figure 3 and Appendix S1: Figure S5) and the initial proportion of hybrids (log‐lmer: β = 1.36, CI = [1.29, 1.43], *p* < 2 × 10^−16^; Figure 3). Exponentiating the model predictors shows that the population size increases by 279% with each additional 10 days of pond duration. A 25% increase in the starting proportion of hybrids resulted in a 40.4% increase in stable population size. When the model was re‐evaluated using scaled predictors, hydroperiod (β = 1.88) was estimated to have a 3.9× greater effect on population size than the starting proportion of hybrids (β = 0.48).

**FIGURE 4:**
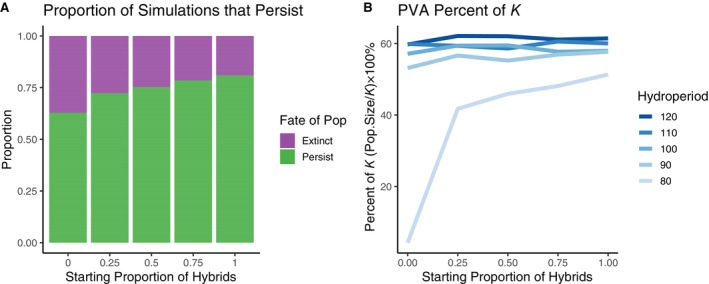
Population viability analysis (PVA) model estimates of population persistence and percent of carrying capacity (*K*) shown across a range of initial hybrid proportion scenarios and hydroperiods. The PVA incorporates environmental stochasticity, which reduces the stable population size below the value for *K* determined in the density‐dependent model. Panel (A) shows the relative proportion of populations that went extinct or persisted across 2500 model iterations. Populations that drop below the quasi‐extinction threshold of three adults during the 100‐year simulation are considered extinct. All simulations that consistently maintain more than three adults are considered to have persisted. Panel (B) depicts the population size as the median number of individuals in the population from years 75 to 100 shown as a percentage of *K*. Lines represent the median percent of *K* across all 500 draws from the posterior distribution of demographic parameter values.

The addition of environmental stochasticity into the demographic model had a large negative impact on population size and persistence. Across all simulations, the population size at the end of the PVA was 58.2% ± 26.2% (median ± SD) of the carrying capacity estimated for each specific scenario (Figure 4 and Appendix S1: Figure S6). Longer hydroperiods significantly increased the population's percent of *K* (log‐lmer: β = 0.086, CI = [0.083, 0.090], *p* < 2 × 10^−16^; Figure 4), as did the proportion of hybrids in the population (log‐lmer: β = 0.823, CI = [0.699, 0.948], *p* < 2 × 10^−16^; Figure 4). The probability of a population persisting to the end of the 100‐year simulation was positively associated with hydroperiod (logit‐glmer: β = 0.406, CI = [0.384, 0.430], *p* < 2 × 10^−16^; Figure 4) and the initial proportion of hybrids (logit‐glmer: β = 4.31, CI = [3.98, 4.66], *p* < 2 × 10^−16^; Figure 4). When the predictors were scaled, a unit increase in hydroperiod (β = 5.75) had a 3.8× greater effect on the probability of persistence than the proportion of hybrids (β = 1.52).

### Hybrid removal simulation

Targeted removal of hybrid salamanders significantly affected the frequency of non‐native alleles in simulated populations: most removal strategies led to a decrease in overall HIS (89.9%) with a few strategies resulting in complete hybrid removal (1.15%), while some led to a detrimental increase in HIS (10.1%), suggesting that optimizing the removal strategy is critical for achieving a desired reduction in hybrids.

**FIGURE 5:**
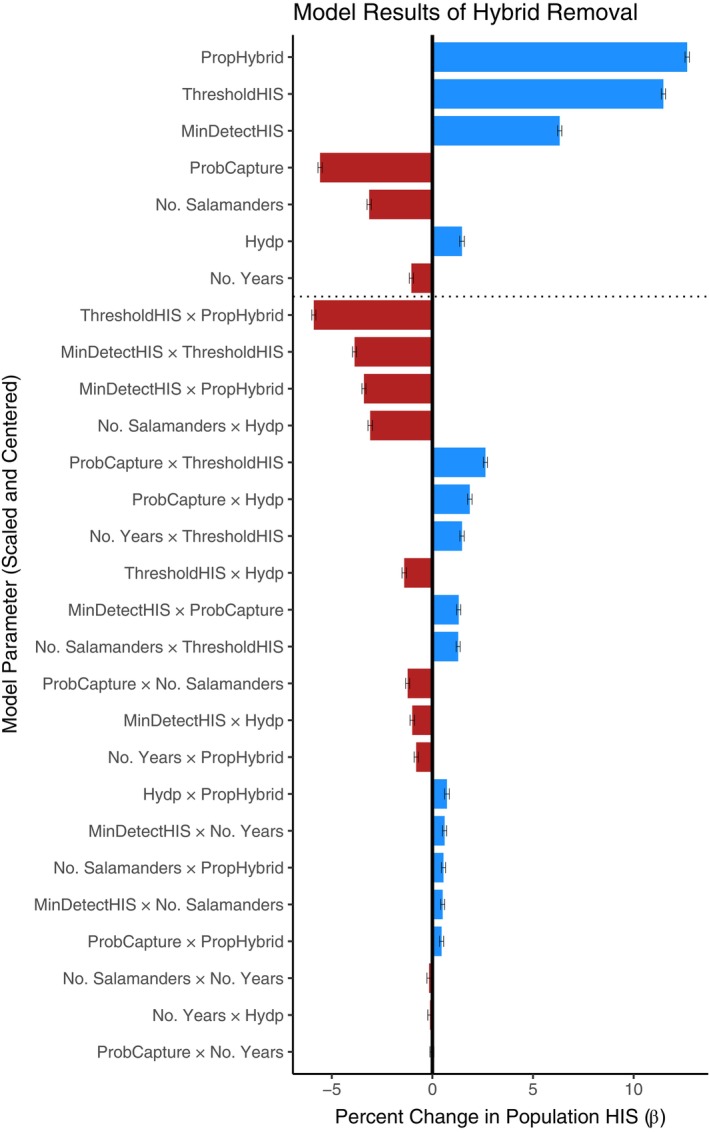
Effect sizes (β) for each parameter included in the hybrid removal simulations. All model parameters were scaled and centered to allow direct comparison between parameters. *X*‐axis shows the slope of each parameter in the generalized linear model. Positive values (blue) indicate an increase in population Hybrid Index Score (HIS) as the parameter value increases, whereas negative values (red) show a decrease in population HIS at the end of the simulation. Each bar shows the magnitude of the effect, with associated black bars indicating the 95% confidence interval for each model parameter.

Each main effect in the generalized linear model (GLM) was significant (Figure 5, and Appendix S1: Table S1 for the complete list), although their effects on HIS varied considerably. Of the main effects, the probability of adult capture (β_scaled_ = −5.58), the number of salamanders screened (β_scaled_ = −3.14), and the number of years of removal (β_scaled_ = −1.04) all had negative slopes, such that increases in any of these parameters resulted in a successful decrease in HIS. The remaining parameters were positively associated with HIS, such that increases in these values would reduce the effectiveness of hybrid removal. These include the initial proportion of hybrids (β_scaled_ = 12.7), hybrid removal threshold (β_scaled_ = 11.5), minimum HIS detectable (β_scaled_ = 6.33), and hydroperiod (β_scaled_ = 1.47). All but one interaction term (“ProbCapture × No. Years”) in the GLM model significantly affected hybrid removal (see Figure 5 and Appendix S1: Table S1).

## DISCUSSION

In this study, we adapted a comprehensive population demographic model constructed for the endangered California tiger salamander (Messerman et al., 2023) to evaluate the potential for hydroperiod management to reduce the prevalence of non‐native hybrids. We used this modeling framework to address three questions critical to CTS management. First, we confirmed that very short hydroperiods (80–90 days) would not support a viable population of CTS (Cooper & Shaffer, 2024). Second, we demonstrated that short hydroperiods can slow, but not reverse, the increase in non‐native alleles. However, hydroperiod can still be used as a tool to promote native CTS and reduce the success of hybrids in many scenarios. Third, using a combination of hydroperiod management and targeted hybrid removal, it is possible to significantly reduce, and even eliminate, hybrids from wild populations. Below, we discuss implementation strategies in specific scenarios to maximize the reduction of non‐native alleles. We believe that these results will be critical for CTS conservation in the face of hybrid introgression and climate change, and suggest that hydroperiod manipulation is a nuanced tool that should be applied differentially depending on local conditions. Although we focus on a single well‐studied system, our approach can also serve as a more general model for the integration of genomic and ecological data into demographic modeling.

### Population growth rate

The density‐independent model confirmed our prediction that populations in longer duration ponds have greater intrinsic growth rates. Based on these results (Figure 1), there appear to be diminishing returns from hydroperiods in excess of 110 days, a pattern that is more pronounced in ponds with a greater proportion of hybrid individuals. In contrast, ponds with an 80‐ to 90‐day hydroperiod have exceptionally low λ estimates, a pattern that is most pronounced in native populations. Based on these results, it would be imprudent to reduce breeding pond duration to less than 90 days for native CTS populations, because this would result in λ less than 1, limiting a population's ability to recover from stochastic events in the short‐term (Kissel et al., 2019; Lennartsson & Oostermeijer, 2001) and ensuring extinction in the long‐term (Lande, 1993).

Our estimate of intrinsic growth rates for native CTS in long hydroperiod conditions was 1.49 (95% CI = 1.28, 1.72), which is nearly identical to Messerman et al. (2023) who reported a λ of 1.48 (95% CI = 1.22, 1.73), suggesting that our modification of the model did not alter its ability to simulate native CTS populations. While both of these values are high, they do not include the strong density‐dependent forces that limit realized growth rates in the field (Trenham & Shaffer, 2005). Hydroperiod has a greater impact on λ than the proportion of hybrids, a result that highlights the importance of long‐duration ponds for native CTS, since it ensures a robust population that can respond quickly to detrimental events. This will likely be important for population persistence in the face of climate change, which is predicted to reduce habitat suitability throughout most of the species' range (Searcy & Shaffer, 2016).

### Carrying capacity

The proportion of hybrids in a population has a large impact on *K*. Our results show that a 25% increase in the initial hybrid proportion results in a 42% increase in *K*. Hydroperiod had an even greater (3.9×) impact, with every additional 10 days increasing the carrying capacity nearly 3‐fold. While hydroperiod has always been highlighted as a critical feature of CTS ecology (Fitzpatrick & Shaffer, 2007a; Johnson et al., 2013), this result quantifies, for the first time, the benefit of longer pond duration on maximum population size. Hydroperiod is an environmental feature that impacts every individual in the population. Longer duration ponds yield higher larval survival rates, resulting in a greater number of metamorphs that partially counteract the density‐dependent reduction in larval survival. This agrees with previous studies on other species of *Ambystoma* that demonstrate the strong influence of hydrology on *K* (Baldwin et al., 2006; McMenamin & Hadly, 2010). While management actions that enhance or restore environmental features that increase *K* (e.g., hydroperiod) are critical for species conservation (Chapman & Byron, 2018), these same modifications will likely benefit hybrids to a greater degree than natives (Cooper & Shaffer, 2024; Fitzpatrick & Shaffer, 2007a; Johnson et al., 2013). Hybrids in these ponds exhibit greater survival, growth, and fecundity than native CTS, increasing both the population size and proportion of hybrids, and resulting in a positive feedback loop that will eventually extirpate native CTS. These increases will also impact surrounding native populations due to increased hybrid dispersal events, or propagule pressure (Semlitsch, 2008; Simberloff, 2009; Trenham et al., 2001).

### Population viability analysis

The addition of environmental stochasticity substantially reduced simulated population sizes to an average of 58% of carrying capacity (*K*). This reduction results from the reliance of successful breeding on hydroperiod, as short ponding durations reduced larval survival (Searcy & Shaffer, 2016), sometimes resulting in complete reproductive failure if ponds dry before any larvae complete metamorphosis (Loredo & van Vuren, 1996; Trenham & Shaffer, 2005). Years with short hydroperiods are also characterized by below‐average precipitation, causing fewer females to emerge to breed and reducing the initial reproductive investment (Trenham et al., 2000; Trenham & Shaffer, 2005). As a result, ponds with short hydroperiods experienced the greatest reduction in population size. While CTS populations do have a remarkable ability to rebound from stochastic decreases, indicated by the relatively high λ of 1.31 across all simulations, the drastic decrease in percent of *K* highlights the importance of environmental variability in CTS life history. This climatic uncertainty may reduce survival and recruitment in any given year, increasing the likelihood of extinction in small, particularly all‐native, populations (Foley, 1994).

The viability of simulated CTS populations was largely driven by hydroperiod. Most populations (70%) with an 80‐day hydroperiod were predicted to go extinct within 100 years (Appendix S1: Figure S6). The low population survival in short‐duration ponds is likely the result of two related processes. First, lower estimates of *K* (seen in the density‐dependent simulations) limit the number of adults in the population even under ideal conditions, rendering them more susceptible to stochastic events (Foley, 1994). Second, low population growth rates (seen in the density‐independent simulations) prevent the population from rebounding after periods of low recruitment (Turkalo et al., 2017), limiting the realized population to a much lower size than *K* (seen in the percent of *K* analysis). This increased time below carrying capacity can increase sensitivity to environmental stochasticity (Lande, 1993). Together, these values result in populations that are unsustainable in 80‐day hydroperiods, corroborating previous evidence that suggests a minimum 90‐day hydroperiod for CTS larval development (Johnson et al., 2013; Petranka, 1998; Stebbins, 2003). In Cooper and Shaffer's (2024) constructed pond hydroperiod experiment, some small CTS did complete metamorphosis in less than 90 days, but this was rare and these stunted individuals would presumably not sustain a population, even without stochastic factors. The 90‐day hydroperiod treatment appears to be the threshold condition that supports a viable population: 36% of simulated populations went extinct over the 100‐year interval. Although a 64% survival probability is unlikely to be an attractive conservation outcome for managers, it may serve as a lower bound of acceptable risk, especially as a temporary condition while hybrid removal strategies are conducted. At 110 days, 94% of all simulated populations, and 87% of all‐native populations, were predicted to persist, which presents a more acceptable long‐term management goal (Figure 4).

### Hybrid removal simulation

Hybrid salamander larvae demonstrate greater fitness than native CTS larvae across a broad spectrum of environmental conditions, including hydroperiod (Cooper & Shaffer, 2024), confirming that hydroperiod management alone cannot reverse hybrid introgression. Since hybrid abatement is specifically required in the USFWS recovery plan for the central CTS population (U.S. Fish and Wildlife Service, 2017), managers will need to employ additional, complementary methods to manage this ongoing threat. This will require genomic surveys to identify cryptic hybrids, so they may be selectively removed from mixed populations. Hybrid removal will require considerable effort, and optimizing the strategy using demographic simulations is vital for efficient implementation. We developed a web‐based shiny application (https://rdcooper408.shinyapps.io/cts_ipm_shiny1/) to enable anyone to run these hybrid removal simulations based on real‐world populations. Below, we list all seven simulated parameters in decreasing order of importance in the hybrid removal model, and describe their relationship with population HIS after 100 years. We describe each main effect and important interactions with other parameters to provide context‐specific recommendations for hybrid removal.

1. *Starting proportion of hybrids*: A greater starting proportion of hybrids in a population limits our ability to reduce HIS, suggesting two important implementation strategies. First, screen populations early and often to identify newly arrived hybrids before they spread (Cooper et al., 2024). Second, prioritize those populations with relatively few hybrids, such as those at the leading edge of the hybrid swarm, to have the greatest return on management investment. Stepping‐stone ponds that connect non‐native and native populations should also be prioritized for removal to prevent further the spread of hybrids, since control and containment is likely to be more effective than eradication, especially in ponds with greater landscape connectivity (Wang et al., 2009).
2. *HIS threshold*: When hybrids are uncommon, the goal should be to remove any hybrid, even those with lower HIS, since it is feasible to completely eradicate hybrids while many native CTS exist to repopulate. However, if the starting proportion of hybrids is high, and only a limited number of individuals can be removed, it is more efficient to be selective and only remove hybrids with higher HIS, as low‐HIS‐hybrids may be the only source of native alleles left in the population. It may be useful to decrease this value over the course of a removal effort, to target individuals with less HIS for removal as the overall population HIS decreases. It is also important to have a sufficient number of genetic markers to accurately determine HIS since our results show that greater precision in assessing HIS improves hybrid removal when using a higher HIS threshold.
3. *Minimum detectable HIS*: More genetic markers (SNPs) used to assess HIS improves hybrid removal. It is critical to employ a sufficient number of SNPs to reliably detect hybrids and quantify HIS, particularly in hybrids with low HIS that may have non‐native alleles present in only 5%–10% of their genome. This precision is especially important in populations with a greater proportion of hybrids and when using a higher HIS threshold for removal. Traditional laboratory genotyping methods using thousands of SNPs are precise but time‐consuming (Cooper & Shaffer, 2024; McCartney‐Melstad et al., 2016); other, less‐precise methods are rapid (Cooper et al., 2024), but still require days to complete. What is needed is further development of rapid genotyping methods that can assay a minimum of 5–10 SNPs in real time in the field.
4. *Probability of capture*: Greater survey efforts are more expensive but will increase the probability of capturing breeding adults in a pond, which significantly improves hybrid removal. The fact that this parameter has a greater model effect than the number of salamanders screened and the number of years of removal makes it the most cost‐effective metric of effort to maximize. However, note that this relationship may change if the breeding population size is much larger than the number of salamanders screened.
5. *Number of salamanders screened*: The more salamanders caught and assayed from a population, the greater the efficacy of hybrid removal. Ideally, any removal strategy would screen all adults captured in the population, although this may not be feasible. Reducing population size, including by reducing hydroperiod, can potentially be used to allow biologists to more easily screen an entire breeding population, and is one example of the synergies that can be achieved between population size and screening strategies.
6. *Hydroperiod*: Longer hydroperiod ponds require greater effort to reduce population HIS. Longer duration ponds increase the number of salamanders in each population (greater *K* and greater % of *K*), requiring increased survey effort and greater numbers of assays to have the same impact on the population. While less effective when deployed on its own, managing hydroperiod may be the most cost‐effective method to increase the impact of hybrid removal and ensure population persistence after removal. For long‐duration ponds in particular, an optimal approach would be to reduce hydroperiod during the hybrid removal activities, then increase hydroperiod after a target HIS, which could include 0, has been reached. Given that most populations rely on artificial livestock ponds, such pond manipulations are feasible. Such a strategy is reversible in time, and could be implemented in collaboration with ranchers to ensure that other above‐ground water troughs provide a more reliable and sanitary source of permanent water for cattle (Willms et al., 2002) that would benefit ranching operations (Bica et al., 2021).
7. *Number of years of removal*: Conducting hybrid removal for a greater number of years results in lower population HIS. While this result is intuitive, it has a surprisingly small relative effect on HIS, and our results suggest that removal for more than 5 years is minimally impactful. However, we note that in our model we assumed a closed system with no hybrid immigration; if a pond is surrounded by other hybrid ponds, longer term monitoring may be critical.

### Caveats and future work

While this study utilizes the best available science to address CTS management concerns, it also has some important limitations. First, some variables in this model have been parameterized using data collected for native CTS (Searcy et al., 2014; Trenham et al., 2000), and those values may differ for hybrids. However, the effects of hydroperiod and genotype on larval survival and mass at metamorphosis, both of which are major factors in CTS demography (Searcy et al., 2014, 2015), were parameterized empirically from hybrids. Second, this study only considers the larval stage of the biphasic life history. Laboratory studies of post‐metamorphic hybrids and CTS (Burger et al., 2024) suggest that temperature‐dependent metabolic differences may also contribute to lifetime fitness differences between hybrids and CTS. Future studies should determine the effect of HIS on metamorph and juvenile/adult growth rates, two variables that have the greatest effect on CTS population growth (Messerman et al., 2023). Our model would also benefit from empirical studies that incorporate the effect of HIS on density‐dependent larval mortality, which affects estimates of carrying capacity in native CTS (Messerman et al., 2023).

We included hydroperiod as a fixed environmental characteristic, which best represents active management conditions. However, variability in the absolute amount and timing of rainfall in unmanaged ponds will affect hydrology. Future work should combine climate projections with rainfall‐dependent hydroperiod models to simulate real‐world pond scenarios and evaluate their expected viability under multiple climate change scenarios. Past modeling has demonstrated that climate change will have a major impact on the range of CTS (Searcy & Shaffer, 2016; Wright et al., 2015), and parameterizing such models for hybrids is an important management need. Our current model also assumes that mating occurs randomly between adults, which results in rapid homogenization of native/non‐native allele frequencies in the population. This is often supported with field data (Cooper & Shaffer, 2024), but we sometimes observe variability in HIS among individuals within ponds and between years (McCartney‐Melstad et al., unpublished data). This pattern may be the result of assortative mating, potentially through behavioral or temporal isolating mechanisms. Finally, we model all diagnostic loci as neutral, when in the wild there is likely a selection gradient acting on CTS/BTS alleles (Cooper & Shaffer, 2021, 2024) which may alter the spread of non‐native alleles in a population. Future studies should investigate these possibilities to improve the accuracy of demographic modeling efforts.

### Management recommendations

Reducing pond hydroperiod alone is insufficient to reverse non‐native introgression. Our results highlight the need to maintain long hydroperiod ponds to support robust native populations. However, ponds that support hybrid populations should be managed to reduce hydroperiod. We therefore suggest the following strategy for reducing non‐native success on the landscape.

1. Survey, genotype, and remove hybrids strategically from ponds characterized by:
  - Recent introgression, often found at the leading edge of the hybrid swarm.
  - High connectivity to neighboring native ponds.
  - Isolated populations unlikely to be recolonized by hybrids.
2. Increase hydroperiod in native populations that are not at risk of hybrid invasion to promote large and robust populations.
3. Decrease hydroperiod in hybrid populations to reduce the success of largely non‐native ponds, while maintaining vernal pool ecological function.
  - Actively manage hydroperiod using adjustable overflow or standpipes.
  - Employ targeted hybrid removal programs.
  - Restore long hydroperiod after hybrid removal to benefit remaining native CTS.
4. Develop rapid detection molecular techniques that could identify hybrids in the field.

### Broader applicability

We provide an example of how to adapt a population‐level demographic model to include individual‐level traits like mass and genotype. Incorporating these characteristics allows us to model complex interactions between genotype, phenotype, and the environment to simulate specific management scenarios, providing clear, actionable recommendations. While we apply this model to native/non‐native hybridization, the approach could be used to incorporate any genotype–environment interaction to understand population dynamics driven by changes in the frequency of adaptive or deleterious alleles. This may be critical for understanding how climate‐adapted loci, which have direct fitness consequences, may affect population persistence and evolution under predicted climate change scenarios. These complex interactions will become increasingly important to guide species recovery in a changing landscape.

## AUTHOR CONTRIBUTIONS

Robert D. Cooper, Arianne F. Messerman, and Christopher A. Searcy contributed to the development of the demographic model. Robert D. Cooper, H. Bradley Shaffer, and Gregory F. Grether guided the development and completion of the project. Robert D. Cooper and H. Bradley Shaffer wrote the manuscript. Robert D. Cooper and Erin Toffelmier completed the statistical analyses and generated the figures. All authors reviewed and edited the manuscript.

## CONFLICT OF INTEREST STATEMENT

The authors declare no conflicts of interest.

## Supporting information


Appendix S1.

## Data Availability

Data are available in Messerman et al. (2022) at https://doi.org/10.5061/dryad.59zw3r291 and Cooper et al. (2024) at https://doi.org/10.1111/cobi.14167. Code (Cooper, 2025) is available in Zenodo at https://doi.org/10.5281/zenodo.16915188.
