# Supplementary material for: Building genomically‐informed demographic models to guide management of invasive hybrids
Source: Ecol Appl. 2025 Oct 23;35(7):e70116. doi: 10.1002/eap.70116 (PMC12550223; doi:10.1002/eap.70116)
Supplement: Supplementary file 1 — Appendix S1. [file EAP-35-e70116-s001.pdf]

Appendix S1

Building genomically-informed demographic models to guide management of invasive hybrids

Robert D. Cooper, Arianne F. Messerman, Christopher A. Searcy, Erin Toffelmier, Gregory F. Grether, H. Bradley Shaffer

Ecological Applications

Integral Projection Model (IPM) Adaptation

We adapted the CTS demographic model constructed by Messerman et al. (2022, 2023). This model combined parameters derived from multiple long term ecological studies on CTS (Trenham et al. 2000; Searcy et al. 2014) to construct a Bayesian IPM for CTS. The model has two distinct stage classes: metamorph and juvenile/adult. Metamorphs begin the season as an aquatic egg, which hatches into an aquatic larva, grows, and then undergoes metamorphosis and transitions to terrestrial life all in the first year. After this first year, all surviving individuals transition to the juvenile/adult class, where they eventually mature into reproductive adults if they survive subsequent years. All of the demographic functions in this model are fit to an individual's mass, the importance of which has been well documented in CTS (Searcy et al. 2014, 2015). Specifically, the IPM uses mass from the previous year to predict the survival probability and the new mass in the present year based on survival and growth functions. It then uses the new mass to estimate maturity, and fecundity for each size class. These functions are fit using long term mark-recapture data collected from two drift fence/pitfall trap studies in Solano and Monterey Counties that spanned 11 and 10 years, respectively.

The original IPM bins each individual by mass into 122 discrete groups. The model then uses two distinct kernels to construct the transition matrix. The first kernel is the product of survival and growth, representing the change in size of an individual if it survived to the next year. A second fecundity kernel estimates the number of offspring an individual would produce given their change in size if they survived to the next year. These two kernels are applied to each stage-class of the model, using separate equations for metamorphs and juvenile/adults. This model uses probability densities for each of the traits and vital rates, and individual variation is not identified in the model. For example, there may be 50 individuals in the 20 gram mass bin, so rather than predicting a new mass for each of the 50 individuals using the growth function, the model uses a probability density of new masses for the entire group simultaneously. A new distribution of masses is created, with no connection between yearly values at the individual level. While this model is useful and efficient for population-level analyses, it does not allow individual traits to transition across years and therefore affect the demographic simulation.

We modified the demographic functions to accept single values for mass and return a single prediction, rather than a probability density. We also restructured the survival, maturity, and breeding probability functions. In the original model these represented probabilities of an event occurring (i.e., 0.2 probability of individuals in this size-bin dying). In our model we used these probabilities to draw a binary response for each individual. We expect that these changes to the IPM will produce more stochasticity in small populations, but that this demographic stochasticity more accurately represents natural conditions and population variation.

Bayesian Demographic Functions

The two functions used to link hydroperiod and the proportion of hybrids were adapted from Cooper and Shaffer (2023). Specifically, we dropped several experiment-specific parameters to improve the generalizability of the model and reduce overfitting for demographic predictions.

Larval Survival

We modeled factors predicting larval survival (*p*) for individual *i* using a hierarchical Bayesian framework. Our likelihood function included pond hydroperiod (HYDP) and larval Hybrid Index Score (HIS). Predictors were all centered and scaled using the SCALE function in R.

$$logit(p_i) = \beta_{HYDP} * HYDP_i + \beta_{HIS} * HIS_i + \beta_0$$
$$y_i \sim Bernoulli(p_i)$$

The prior distribution for β_0 was specified as the logit-transformed probability of survival (p_0), and the prior distribution for p_0 was a beta binomial distribution with parameters α = 1 and β = 1. A uniform prior distribution from -5 to 5 was used for all slope parameters β_{HYDP}, β_{HIS}. The model was iterated 500 times to explore the variation present in the larval resampling process. We combined the posterior distributions from each of the 500 iterations to estimate model parameters.

Metamorph Mass

We modeled individual metamorph mass ($MASS_i$) as:

$$\mu_i = \beta_{HYDP^2} * HYDP_i^{\ 2} + \beta_{HYDP} * HYDP_i + \beta_{HIS} * HIS_i + \beta_0$$
$$MASS_i \sim Normal(\mu_i, \tau)$$

The prior distribution for β_0 was set to a normal distribution with hyperpriors mean (μ_i) and standard deviation (τ) which were shared across treatment levels. The prior for μ_i was set as a normal distribution with a mean of 0 and standard deviation of 0.1. We used a uniform distribution from 0 to 10 as the prior for variance (σ), and precision (τ) was then computed as $\frac{1}{\sigma^2}$. A uniform distribution was used for all slope parameters with a range: from -30 to 0 for β_{HYDP^2}, from 0 to 30 for β_{HYDP}, and from -10 to 10 for β_{HIS}. These distributions were updated to ensure that the estimates were not restricted based on the prior.

MCMC Implementation

For all Bayesian analyses we used the R package JAGSUI (version 1.5.2), which implements a Gibbs Sampler in the R environment. Unless otherwise indicated, all Bayesian models were iterated 10,000 times with four independent chains. We allowed automatic adaptation and specified zero-iteration burn-in. Bayesian model convergence was assessed using the Potential Scale Reduction Factor (PSRF or Rhat [$\hat{R}$]), which we required to be less than 1.05. We visually inspected the MCMC output using the R package MCMCOUTPUT (version 0.1.1) to ensure adequate convergence and normal posterior distributions. Parameter significance was determined if the 95% Credible Interval (CI) did not include zero. Parameter estimates were taken as the mean value of the posterior distribution.

Further description of BTS detection threshold

BTS detection threshold (values simulated: 0.1 to 0.5 in increments of 0.1) is the average proportion of the genome that must contain non-native alleles for the detection assay to correctly identify an individual as hybrid. While correlated, this is not the same as HIS, since it depends on how those alleles are distributed across the genome (i.e., zygosity). For example, if 20% of loci had non-native alleles in the homozygous condition, the HIS would be 0.20. However, if 20% of loci were heterozygous (CTS/BTS) then it's HIS would be 0.10. BTS detection threshold can also be conceptualized as the inverse number of SNPs assayed when genotyping salamanders: 4 SNPs would allow you to detect a hybrid with BTS alleles in as few as 25% of its loci (HIS between 12.5-25%), whereas 10 SNPs would allow you to detect hybrids with BTS alleles in as few as 10% of its loci (HIS between 5% - 10%). Note that this is a very rough approximation with extremely coarse resolution.

Supplemental Tables:

	Slope β	LowerCI	UpperCI
(Intercept)	-28.1	-27.9	-28.2
Proportion_Of_Hybrids	12.7	12.8	12.6
HIS_Threshold_For_Removal	11.5	11.6	11.4
BTS_Detection_Threshold	6.33	6.43	6.22
Probability_Of_Adult_Capture	-5.58	-5.48	-5.69
Max_N_Sal_Screened	-3.14	-3.04	-3.25
Hydroperiod	1.47	1.58	1.35
N_Years_Hybrids_Removed	-1.04	-0.940	-1.15
HIS_Threshold_For_Removal * Proportion_Of_Hybrids	-5.89	-5.79	-5.99
BTS_Detection_Threshold * HIS_Threshold_For_Removal	-3.87	-3.77	-3.97
BTS_Detection_Threshold * Proportion_Of_Hybrids	-3.40	-3.30	-3.50
Max_N_Sal_Screened * Hydroperiod	-3.09	-2.98	-3.20
Probability_Of_Adult_Capture * HIS_Threshold_For_Removal	2.63	2.74	2.53
Probability_Of_Adult_Capture * Hydroperiod	1.86	1.97	1.74
N_Years_Hybrids_Removed * HIS_Threshold_For_Removal	1.47	1.58	1.36
HIS_Threshold_For_Removal * Hydroperiod	-1.40	-1.29	-1.51
BTS_Detection_Threshold * Probability_Of_Adult_Capture	1.31	1.41	1.21
Max_N_Sal_Screened * HIS_Threshold_For_Removal	1.28	1.38	1.18
Probability_Of_Adult_Capture * Max_N_Sal_Screened	-1.23	-1.13	-1.33
BTS_Detection_Threshold * Hydroperiod	-1.00	-0.888	-1.11
N_Years_Hybrids_Removed * Proportion_Of_Hybrids	-0.804	-0.703	-0.906
Hydroperiod * Proportion_Of_Hybrids	0.722	0.841	0.604
BTS_Detection_Threshold * N_Years_Hybrids_Removed	0.605	0.710	0.500
Max_N_Sal_Screened * Proportion_Of_Hybrids	0.551	0.652	0.449
BTS_Detection_Threshold * Max_N_Sal_Screened	0.512	0.610	0.413
Probability_Of_Adult_Capture * Proportion_Of_Hybrids	0.457	0.560	0.354
Max_N_Sal_Screened * N_Years_Hybrids_Removed	-0.165	-0.0577	-0.272
N_Years_Hybrids_Removed * Hydroperiod	-0.118	-0.00587	-0.230
Probability_Of_Adult_Capture * N_Years_Hybrids_Removed	-0.0218	0.0786	-0.122

Table S1: Model results from hybrid removal simulations. Table shows slopes (β) derived from the GLM model that evaluated the percent change in population hybrid index score (HIS) after 100 years based on multiple hybrid removal parameters. Negative slopes indicate beneficial decreases in population HIS, while positive slopes indicate a relative increase in HIS. We developed a web-based shiny application (https://rdcooper408.shinyapps.io/cts_ipm_shiny1/) to enable anyone to run these hybrid removal simulations based on real-world populations.

Figures

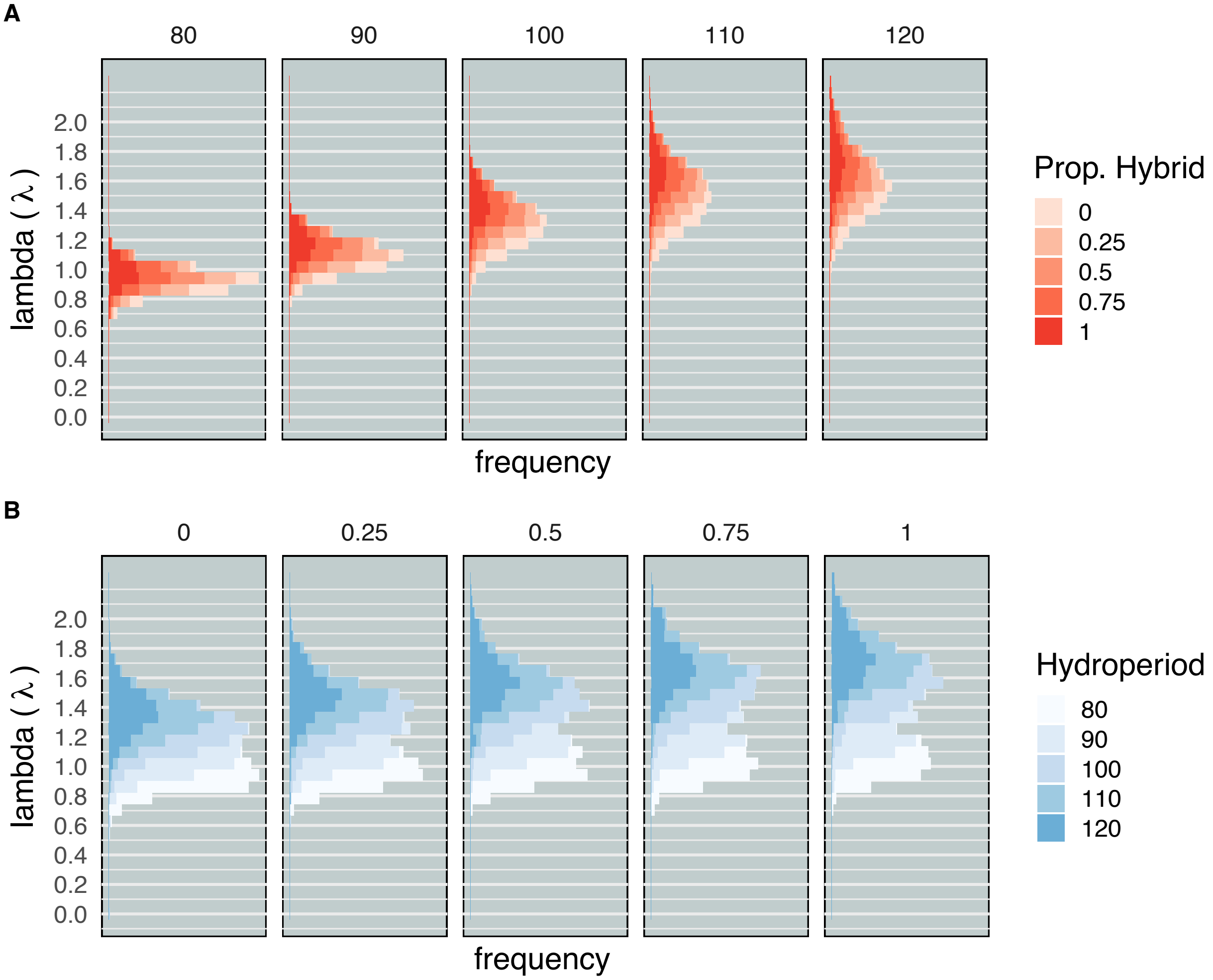

Figure S1: Density-independent model estimates of population growth rate (Lambda or λ) across demographic scenarios. Lambda is estimated as the slope of the log-normalized adult population size and time (years), after an initial burn-in of 2 years. The simulation was initiated with different combinations of pond hydroperiod (A) and the starting proportion of hybrid individuals (B) in the population. Longer hydroperiods and higher hybrid frequencies yield greater λ estimates. Short hydroperiods and more native populations result in lower values for λ, some of which are less than 1, indicating a declining population.

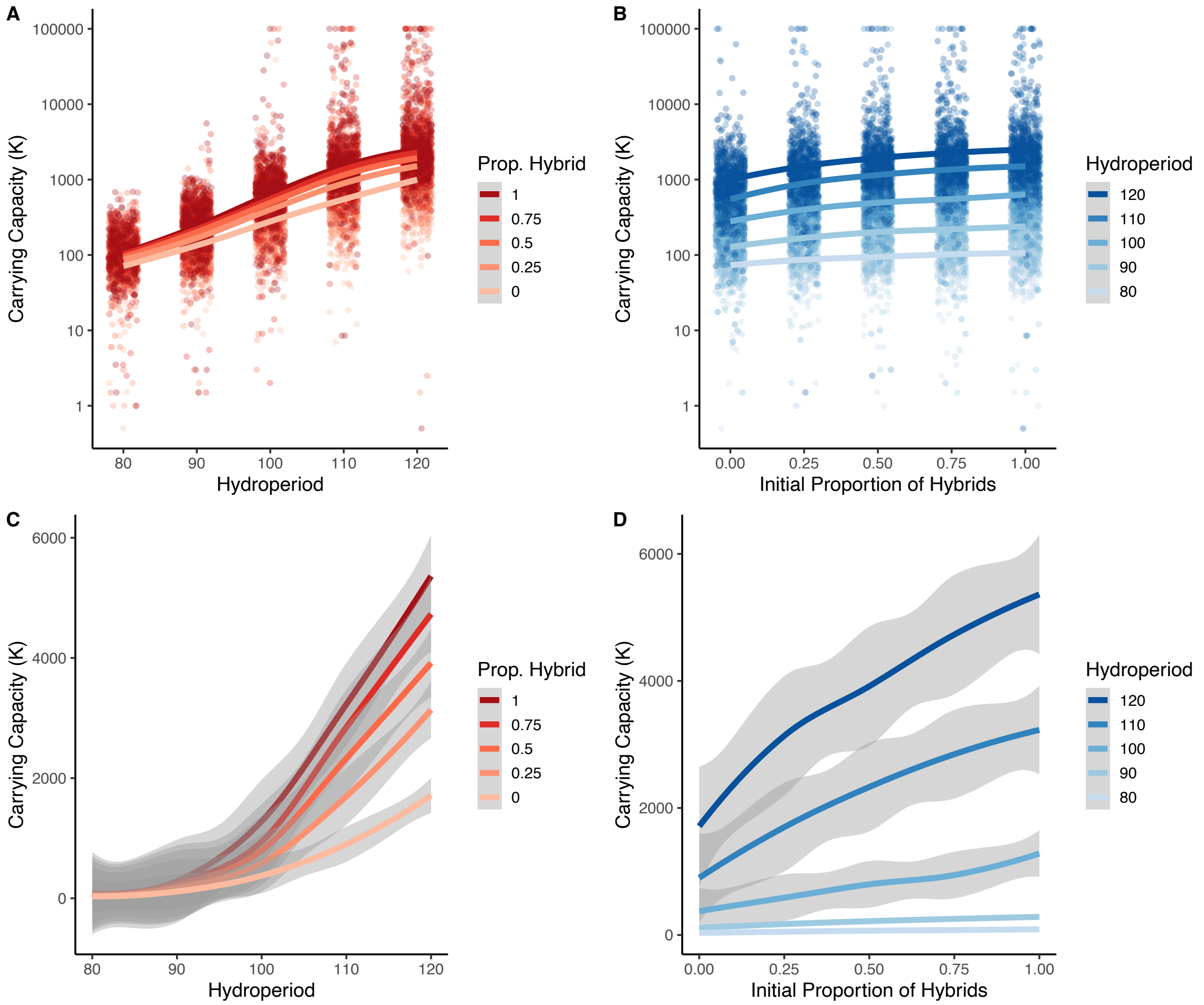

Figure S2: Density-dependent model estimates for carrying capacity (K) across a range of pond hydroperiods (panels A, C) and starting proportion of hybrids (panels B, D). Panels show the median population size from years 75 to 100 of the 100-year simulations. The first 75 years are removed to allow the populations sufficient time to reach their stable equilibrium. Colored lines represent locally estimated scatterplot smoothing (loess) model predictions with standard error depicted as grey ribbons. Panels A and B are plotted using a base 10 logarithmic scale to improve resolution, while panels C and D are plotted on a linear scale.

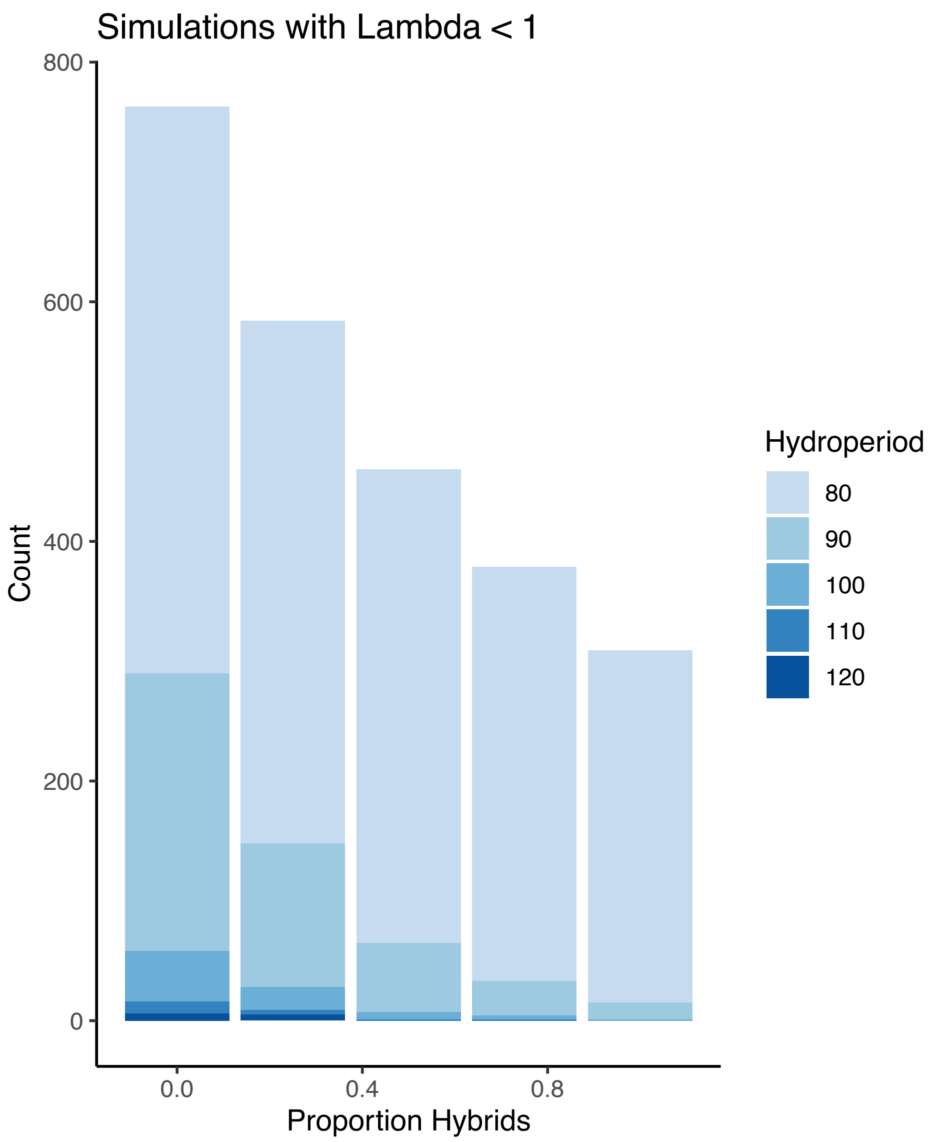

Figure S3: Distribution of density-independent simulations with Lambda less than 1.

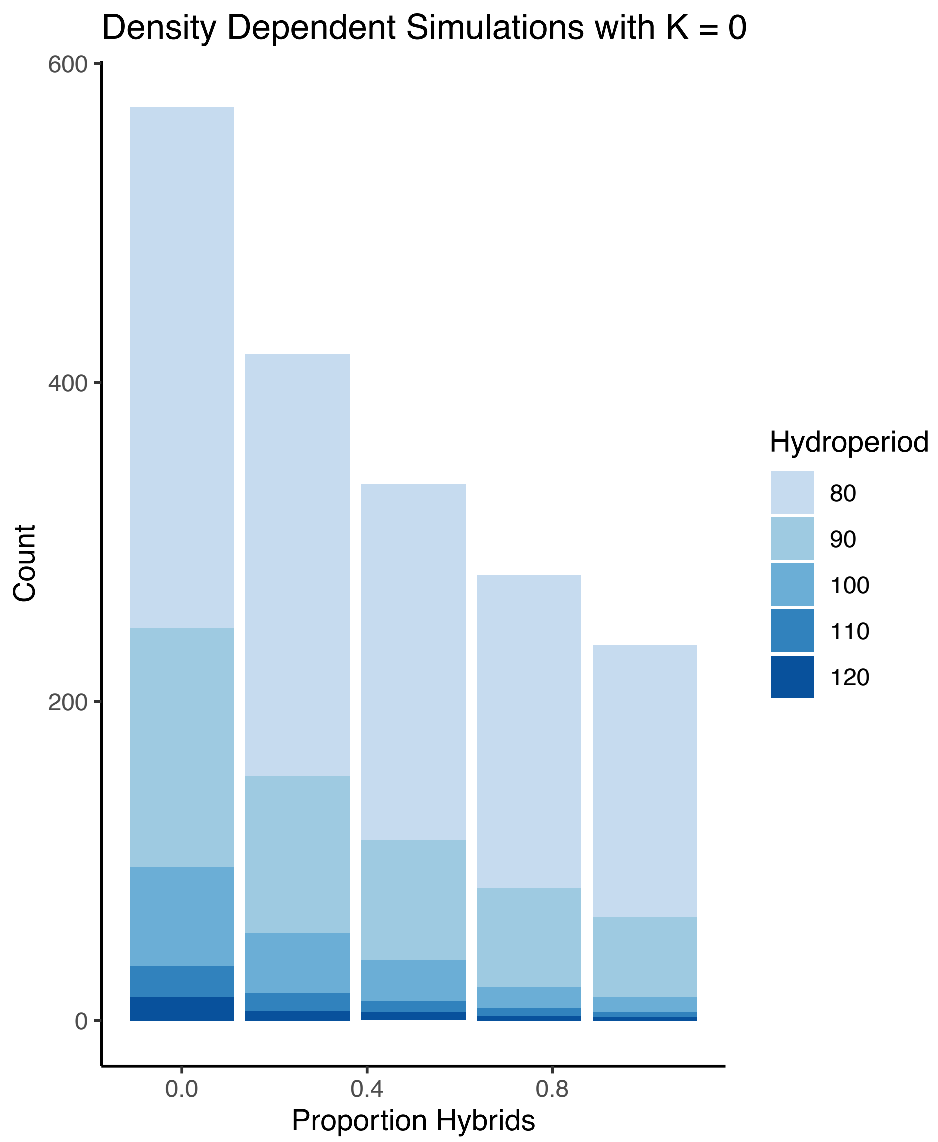

Figure S4: Distribution of density-dependent simulations with carrying capacity equal to 0, which represents populations that went extinct.

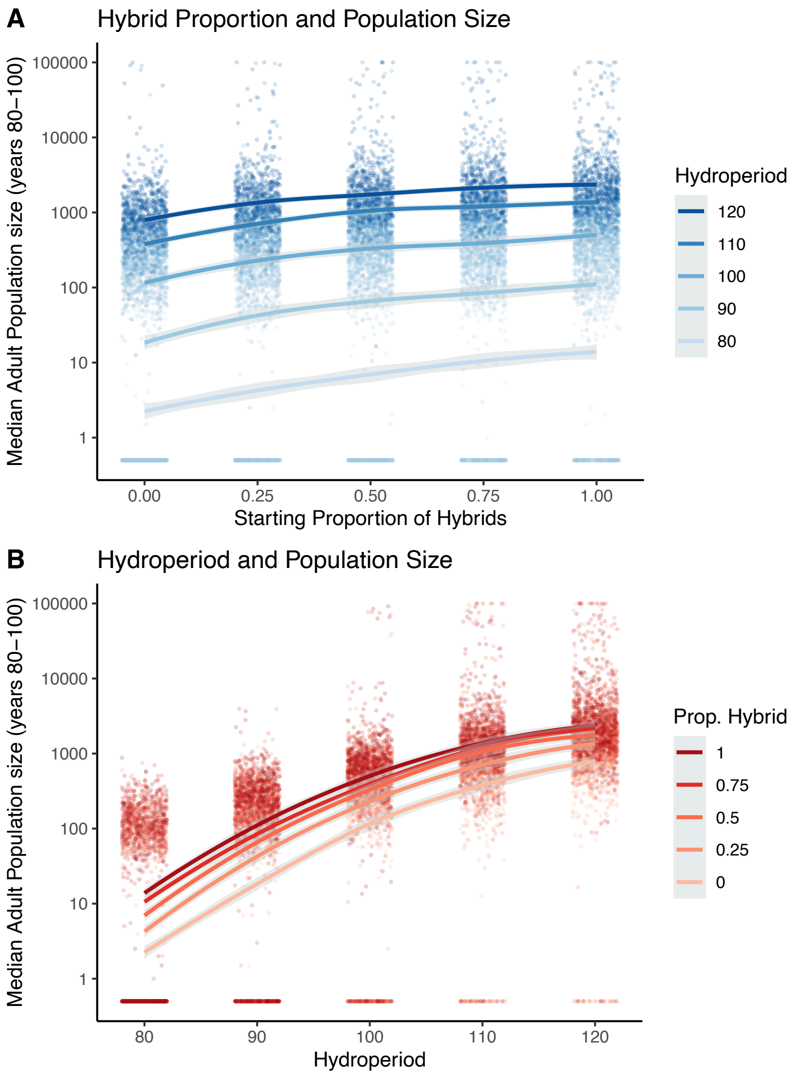

Figure S5: Population Viability Analysis (PVA) model estimates of stable population size. Population size is calculated as the median number of adults in the population in years 80 - 100. The PVA model includes environmental stochasticity, which yields new estimates for equilibrium population size. We show the estimated median population size with respect to initial hybrid proportion (A) and hydroperiod (B) simulated across 500 draws from the posterior distributions of all demographic functions. Population size is shown on a logarithmic scale to increase the resolution of low population-size scenarios. Horizontal lines at the bottom of each panel represent populations that have gone extinct.

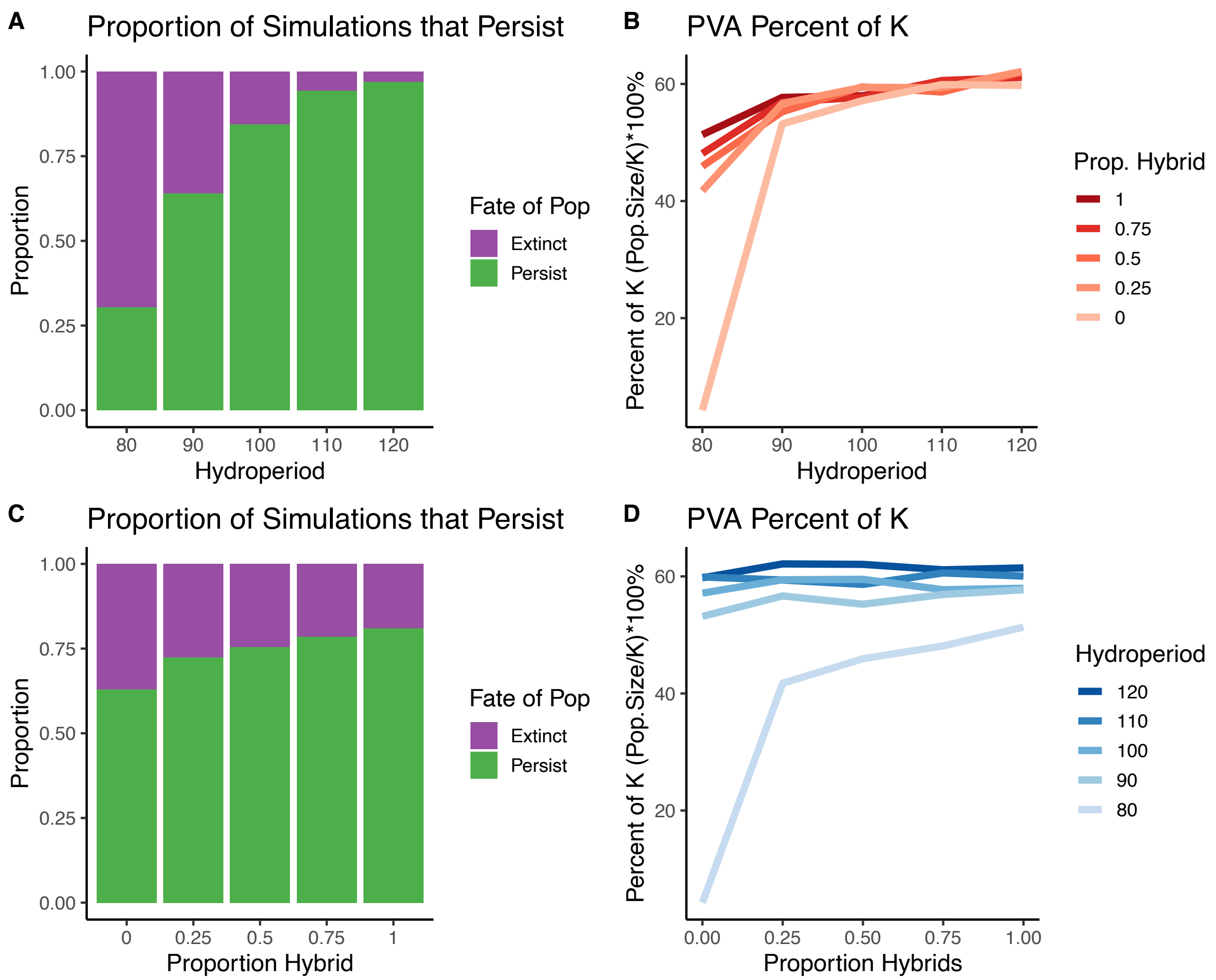

Figure S6: Population Viability Analysis (PVA) model estimates of population persistence and percent of carrying capacity (K) shown across a range of hydroperiod (panels A, B) and initial hybrid proportion (panels C, D) scenarios. The PVA incorporates environmental stochasticity, which reduces the stable population size below the value for K determined in the density-dependent model. Panels A and C show the relative proportion of populations that went extinct or persisted across 2500 model iterations. Populations that drop below the quasi-extinction threshold of 3 adults during the 100-year simulation are considered extinct. All simulations that consistently maintain more than 3 adults are considered to have persisted. Panels B and D depict the population size as the median number of individuals in the population from years 80-100 shown as a percentage of K. Lines represent the median percent of K across all 500 draws from the posterior distribution of demographic parameter values.

Literature Cited:

Messerman A, Clause A, Gray L, Krkošek M, Rollins H, Trenham P, Shaffer B, Searcy C. 2022, October 21. Data: Applying stochastic and Bayesian integral projection modeling to amphibian population viability analysis. Dryad. Available from https://datadryad.org/stash/dataset/doi:10.5061/dryad.59zw3r291 (accessed February 6, 2024).

Messerman AF, Clause AG, Gray LN, Krkošek M, Rollins HB, Trenham PC, Shaffer HB, Searcy CA. 2023. Applying stochastic and Bayesian integral projection modeling to amphibian population viability analysis. Ecological Applications **33**:e2783.

Searcy CA, Gray LN, Trenham PC, Shaffer HB. 2014. Delayed life history effects, multilevel selection, and evolutionary trade-offs in the California tiger salamander. Ecology **95**:68–77.

Searcy CA, Snaas H, Shaffer HB. 2015. Determinants of size at metamorphosis in an endangered amphibian and their projected effects on population stability. Oikos **124**:724–731.

Trenham P, Shaffer BH, Koenig W. 2000. Life history and demographic variation in the California tiger salamander (Ambystoma californiense).
